# Numerical investigation of transient mixed convection of nanofluid in a cavity with non-Darcy porous inner block and rotating cylinders with harmonic motion

**DOI:** 10.1038/s41598-021-96733-6

**Published:** 2021-08-26

**Authors:** Nima Shirani, Davood Toghraie

**Affiliations:** Department of Mechanical Engineering, Khomeinishahr Branch, Islamic Azad University, Khomeinishahr, Iran

**Keywords:** Engineering, Mathematics and computing, Nanoscience and technology

## Abstract

Mixed convection of nanofluid in a 2D square enclosure with a porous block in its center and four rotating cylinders, which are forced by a simple harmonic function, was studied numerically. The porous zone was studied by considering the Forchheimer–Brinkman-extended Darcy model. Effects of various parameters including Darcy number (10^–5^ ≤ Da ≤ 10^–2^), porosity (0.2 ≤ ɛ ≤ 0.7), Richardson number (0.1 ≤ Ri ≤ 10), and volume fraction of nanoparticles (0 ≤ ϕ ≤ 0.03), on heat transfer, entropy generation, PEC, velocity, streamline and isotherm contours were demonstrated. The results show that decreasing the Darcy number as well as reducing the Richardson number leads to an increase in the average Nusselt number. However, porosity changes had no decisive effect on heat transfer. Maximize the volume fraction of copper nanoparticles in the base fluid enhanced heat transfer. In the case of the high permeability of the porous medium, the impact of the harmonic rotation of the cylinders on the flow patterns became more pronounced.

## Introduction

The heat transfer mechanism has a pivotal effect on design of industrial equipment. Hence, engineers and researchers have always strived to improve them. There are many ways to upgrade these systems, and among them adding nanoparticles to base fluids has a decisive effect on improving the heat transfer rate. Furthermore, employing external gears to increase the fluid velocity has a marginal effect on heat transfer rate. By analyzing and optimizing these factors in different geometries and problems, their productivity can be increased. Numerical simulations are a much-needed help in analyzing and predicting different phenomena in different geometries; hence, many researchers employed this method to investigate both industrial and theoretical problems. The convection of fluid flow under the diverse condition in the enclosures is a grave problem with various applications such as solar panels, electronic modules, indoor thermal conditions, etc. These types of problems were studied by a myriad of scholars and researchers. Kladias et al.^1^ verified the Darcy–Brinkman–Forchheimer flow model for natural convection in porous media experimentally. Their results show that Darcy's number has a direct relationship with heat transfer rate. Also, by decreasing Darcy's number from 10^–2^ to 10^–6^, the Nusselt number increases by 65.321%. Moreover, they found that the influence of thermal conductivity was deemed to be more evident at low Darcy and Rayleigh numbers. Lai et al.^2^ investigated mixed convection in a vertical wall with porous media. They found that by considering heat dissipation, heat transfer rate was enhanced and thermal and hydrodynamic boundary layers were become thicker. Baytas et al.^3^ investigated free convection in a cavity including a heated solid phase, thermal non-equilibrium non-Darcy porous media. They found that for a low thermal conductivity ratio, the difference between fluid and solid temperatures was at its highest level. Al Zahrani et al.^4^ performed an analysis of the mixed convection in the porous space between twain concentric cylinders. The obtained results show that if the thermal conductivity ratio is further than its critical value, the average Nusselt number decreases. Sivasankaran et al.^5^ studied mixed convection in a porous cavity. They realized that non-uniform heating has a positive effect on heat transfer rate. Furthermore, by increasing Darcy number and porosity, the heat transfer rate was enhanced. Bera et al.^6^ investigated double-diffusive natural convection within a porous square cavity. They discovered that for low permeability case, the results of the Darcy model were not much diverse from that of non-Darcy. Nguye et al.^7^ studied free convection in the porous cavity using the characteristic-based splitting method. They figured out that at high Rayleigh number and low Darcy numbers, the effect of the addition of the nanoparticles was reduced. Astanina et al.^8^ analyzed temperature-dependent viscosity in a square porous enclosure. They found that the viscosity variation parameter has a direct relationship with heat transfer rate. Investigation on mixed convection of CuO-Water nanofluid in a cavity with porous medium and an adiabatic rotating cylinder was performed by Selimefendigil et al.^9^. Their results show that the effect of increasing the rotational velocity of the cylinder on heat transfer was more obvious for larger cylinder diameters. Rajarathinam et al.^10^ adopted the Darcy–Brinkman–Forchheimer model to examine the impact of the presence of nanofluid in an inclined porous cavity. Their results demonstrate that the direction of the moving wall is the most essential factor for the variation of the flow pattern and heat transfer rate. Alsabery et al.^11^ studied entropy generation and heat transfer rate in a porous cavity with wavy boundaries and a rotating cylinder. Their results show that increasing porosity leads to increasing Nusselt number and decrease Bejan number. Maghsoudi et al.^12^ analyzed mixed convection of a nanofluid in a heterogeneous porous cavity with two lid-driven sides. They optimized pore size arrangement at different parts of the cavity and found that the optimized heterogeneous porous medium was capable to augment heat transfer rate for the low and high value of Richardson number up to 8.3% and 0.9%, respectively. Mehmod et al.^13^ studied the impact of MHD on energy diffusion through ferrofluid in a trapezoidal container with a porous medium. They found that the effect of conduction on ferrofluid flow was more pronounced at low Hartmann numbers. Siavashi et al.^14^ investigated the impact of jagged wedge-shaped porous media on heat transfer rate. They reported that the Euler number was lower for nanofluid in comparison with bas fluid at higher Reynolds numbers. Sheikholeslami et al.^15^ numerically studied energy transport of ferrofluid in wavy wall porous cavity with radiation and EHD effects. They found that fluid circulation was much more affected by increasing supplied voltage reflects than the increase in the Darcy number. Bondarenko et al.^16^ analyzed the impact of the two-centered porous block on heat transfer of Al_2_O_3_-water nanofluid flow in a cavity. They reported that by expanding porous block size, the heat conduction effects were increased and cooling was not performed efficiently in the enclosure. Dogonchi et al.^17^ studied the impact of inclined magnetic fields on the natural convection of nanofluids in a porous gap between a rectangular and circular cylinder. They presented that increasing the angle of the magnetic field did not have a favorable effect on heat transfer and this behavior was more noticeable at higher Darcy numbers. Mikhailenko et al.^18^ performed a numerical study on natural convection in a cavity with uniform rotation and a porous layer. They found that the stream function was reduced by increasing the thickness of the porous layer. Raizah et al.^19^ studied a V-shaped cavity filled with nanofluid and heterogeneous porous medium using the ISPH scheme. They found that the size of the boundaries, which are considered cold and hot walls, have a magnificent effect on the hydro-thermal behavior of the cavity. Ahmed et al.^20^ investigated entropy generation of porous media and nanofluid in an odd-shape cavity. They located the porous medium with altered shapes in a different part of the cavity. They reported that heterogeneous porous medium had a direct effect on irreversibility. Guerrero et al.^21^ numerically studied unsteady convection of solute in a porous cavity. Their results show that the Rayleigh number had a decisive role on the properties of the permeable zone. Convection in an inclined rectangular cavity filled with the permeable medium was investigated by Guerrero et al.^22^. They found that increasing slope angle decreases Nusselt number. The stable convection modes are highly related to the aspect ratio of their considered cavity. Guerrero et al.^23^ numerically investigated natural convection in three layers permeable medium of a 3D cavity. Their main conclusion demonstrates the remarkable effect of thermal conductivity of solid zone. Moreover, the three tested porous layers' thickness has a minor effect on Nusselt number for different permeability ratios. Qi et al.^24^ experimentally investigated the presence of the metal foam saturated with Fe_3_O_4_-water nanofluid in a cavity under a magnetic field. They also studied the effects of different rotation angles of the cavity. They found that the Nusselt number was enhanced by 11.29% for the case with a rotation angle equal to 90 degrees; moreover, increasing PPI (Pores per Inch) leads to a decrease of Nusselt number. Qi et al.^25^ used the lattice Boltzmann model to study the natural convection of Cu-water nanofluid in solar collectors. In addition to examining the effect of porous medium on heat transfer, many researchers^19–23,26–51^ examined the influence of different parameters on the performance of industrial equipment. In general, they considered the shear stress due to the presence of a rotating body in the fluid as an important factor in disrupting flow patterns and changing the thermal behaviors of the fluid. Increasing the heat transfer rate was reported as a result of the presence of a rotating cylinder, but its optimization was the subject of recent research. Recent studies were shown that the direction of rotation of the cylinder, the size of the cylinder, its location in any type of displacement are highly influential factors; However, few studies were examined all of these parameters plus the effect of the number of cylinders for each angle of the square cavity. To examine this purpose more specifically and accurately, in previous works, we^36,50^ numerically studied the presence of four cylinders with a harmonic rotation in terms of the size of the cylinders, their position, and the direction of their rotation as a number. In this study, by selecting the best size and position of the cylinders from our previous articles, we investigate the effects of porous blocks on the mixed convection of nanofluid flow inside the square cavity with rotating cylinders. Permeable medium block helps to control the flow patterns as well as temperature distribution inside the cavity. To investigate this issue, the results are presented and carefully reviewed below.

## Problem statement

**Figure 1:**
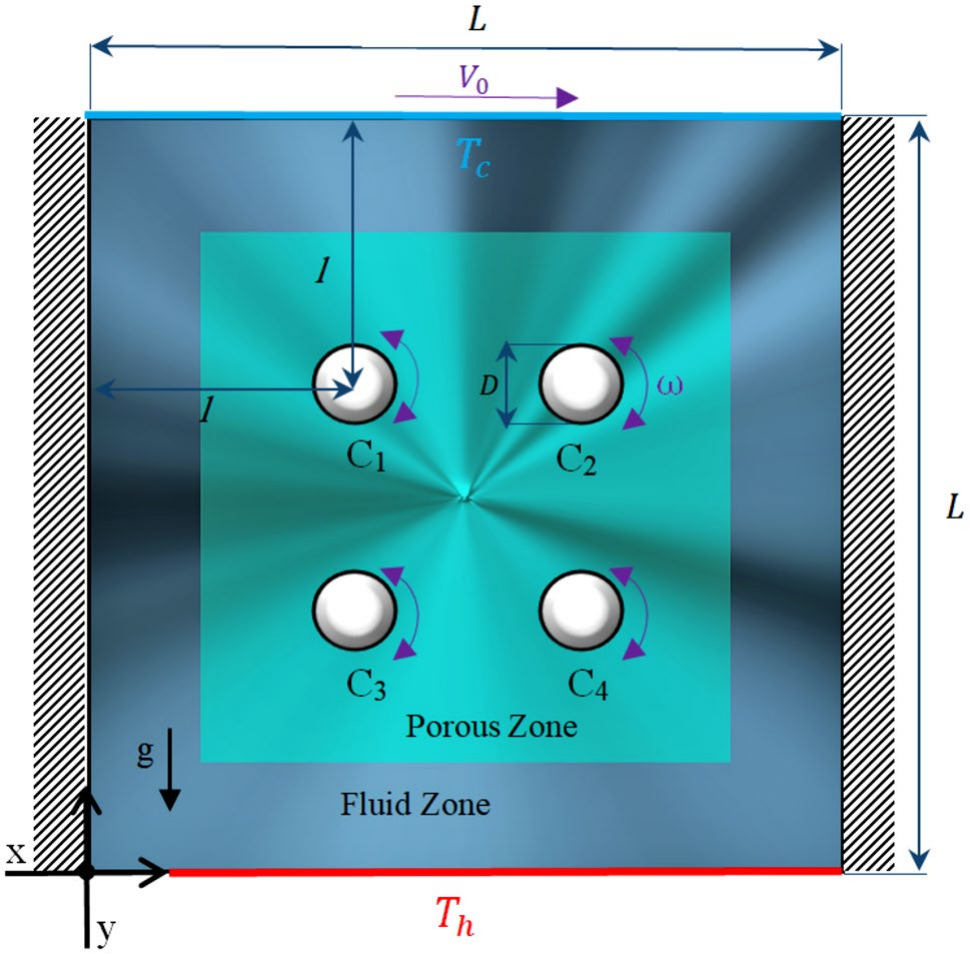
Schematic of the physical model.

Figure 1 illustrates a schematic of a 2D cavity with a height and length of L and a centered porous block with a height and length of 0.7 × L. The bottom wall and top lid-driven wall are considered with hot and cold temperature, respectively and other walls are adiabatic. Four cylinders, which have fixed diameter D = 0.1 × L and distance from the center of the cylinder to the sidewall l = 0.35 × L, are rotating under a harmonic function. Dimensionless angular velocity Ω = 5 was used for cylinder rotational velocity for the flow velocity to be affected by the Richardson number and the detection of natural, mixed, and forced convection to be more accurate. The Cu-water nanofluids were assumed Newtonian, incompressible, and laminar with constant thermophysical properties (Table 1) except density which needs to apply Boussinesq approximation. Grashof number Gr = 74,363.3974 was used to determine the ratio of buoyancy force to viscous force. It is worth mentioning that both the thermal conductivity of the solid zone and temperature differences between solid and fluid zone were allowed us to not use the LTNE model.

**Table 1:** Thermophysical properties of water and copper nanoparticles^36^.

Physical properties	Water	Cu
${C}_{p}$ (J/Kg K)	4179	385
$\rho$ (Kg/m^3^)	997.1	8933
$\mu$ (W/m K)	9.09 × 10^–4^	–
k (W/m K)	0.613	401
$\beta$ (K^−1^)	21 × 10^–5^	1.67 × 10^–5^

## Mathematical formulation

The continuity, momentum, and energy conservation equations in dimensional form are as follows^10^:

1 $$\nabla . \mathbf{u}=0$$

2 $$\frac{1}{\varepsilon }\frac{\partial {\varvec{u}}}{\partial t}+\left(\frac{{\varvec{u}}}{{\varepsilon }^{2}}\right) . \nabla \mathbf{u}=-\frac{1}{{\rho }_{nf}}\nabla p+\frac{{\nu }_{nf}}{\varepsilon }{\nabla }^{2}{\varvec{u}}-\frac{{\nu }_{nf}}{K}{\varvec{u}}-\frac{Fc}{\sqrt{K}}{\varvec{u}}\left|{\varvec{u}}\right|+g{\beta }_{nf}\left(T-{T}_{c}\right)$$

3 $$\sigma \frac{\partial T}{\partial t}+{\varvec{u}} . \nabla T=\frac{{k}_{m}}{{\left(\rho {C}_{p}\right)}_{nf}}{\nabla }^{2}T$$

where **u** and ɛ are the Darcy velocity and porosity, respectively.

The permeability and Forchheimer coefficient are defined by Ergun equation^52^:

4 $$K=\frac{{\varepsilon }^{3}{d}_{p}^{2}}{a{\left(1-\varepsilon \right)}^{2}}$$

5 $$F=\frac{b}{\surd a{\varepsilon }^\frac{3}{2}}$$

6 $$\sigma =\frac{\varepsilon {\left(\rho {C}_{p}\right)}_{nf}+(1-\varepsilon ){\left(\rho {C}_{p}\right)}_{s}}{{\left(\rho {C}_{p}\right)}_{nf}}$$

7 $${k}_{m}=\left(1-\varepsilon \right){k}_{s}+\varepsilon {k}_{nf}$$

where d_p_ is the average particle size of the bed and Ergun constants are a = 150 and b = 1.75 and $\sigma =1$ and ${k}_{nf}={k}_{s}$. Therefore, the final form of Eq. (3) is^10^:

8 $$\frac{\partial T}{\partial t}+\left(u\frac{\partial T}{\partial x}+v\frac{\partial T}{\partial Y}\right)=\frac{{k}_{nf}}{{\left(\rho {C}_{p}\right)}_{nf}}{\nabla }^{2}T$$

The following equations are dimensionless parameters in this study:

9 $$Da=\frac{K}{{L}^{2}}$$

10 $$\mathrm{Re}={V}_{0}\mathrm{L}/{\upupsilon }_{nf}$$

11 $$\mathrm{Ri}=Gr/{Re}^{2}$$

12 $$\mathrm{Gr }=\mathrm{g }{\upbeta }_{nf} ({T}_{h}-{T}_{c}){L}^{3}/ {\upupsilon }_{nf}^{2}$$

13 $$\mathrm{Pr}={\left({C}_{p}\mu \right)}_{nf}/{k}_{nf}$$

14 $$\Omega =\upomega /(2.{V}_{0}/d)$$

15 $$\uptau =t {\mathrm{\alpha }}_{nf}/{L}^{2}$$

16 $$PEC=\frac{{Nu}_{ave}/({{Nu}_{ave})}_{\phi =0}}{({{Cf}_{ave}/{{Cf}_{ave}}_{\phi =0})}^{1/3}}$$

17 $${C}_{f}=\frac{{\tau }_{w}}{\frac{1}{2}\rho {{V}_{0}}^{2}}$$

18 $${{C}_{f}}_{ave}=-\frac{1}{L}{\int }_{0}^{L}{C}_{f,x}dx$$

19 $$\uptheta =(T-{T}_{c)}/\left({T}_{h}-{T}_{c}\right)$$

20 $$\mathrm{V}=v/{V}_{0}$$

21 $$\mathrm{U}=u/{V}_{0}$$

22 $$\mathrm{Y }=y/L$$

23 $$\mathrm{X }=x/L$$

24 $$\mathrm{P}=\frac{p}{{\rho }_{nf}{V}_{0}^{2}}$$

25 $${\uppsi }^{*}=\frac{\uppsi }{{\mathrm{\alpha }}_{nf}}$$

26 $${\omega }^{*}=\frac{\overrightarrow{\omega }}{{\mathrm{\alpha }}_{nf}{/L}^{2}}$$

The dimensionless forms of Eqs. (1)–(3) are as follows^10^,

27 $$\frac{\partial U}{\partial \mathrm{X}}+\frac{\partial V}{\partial X}=0$$

28 $$\frac{1}{\varepsilon }\frac{\partial U}{\partial \tau }+\frac{U}{{\varepsilon }^{2}}\frac{\partial U}{\partial X}+\frac{V}{{\varepsilon }^{2}}\frac{\partial U}{\partial Y}=-\frac{\partial P}{\partial X}+\frac{1}{\upvarepsilon }\frac{{\nu }_{nf}}{{\nu }_{f}}\frac{1}{Re}{\nabla }^{2}U-\frac{{\nu }_{nf}}{{\nu }_{f}}\frac{1}{Re}\frac{U}{Da}-\frac{Fc}{\sqrt{Da}}U\sqrt{{U}^{2}+{V}^{2}}+\frac{{\beta }_{nf}}{{\beta }_{f}}Ri\theta$$

29 $$\frac{1}{\varepsilon }\frac{\partial V}{\partial \tau }+\frac{U}{{\varepsilon }^{2}}\frac{\partial V}{\partial X}+\frac{V}{{\varepsilon }^{2}}\frac{\partial V}{\partial Y}=-\frac{\partial P}{\partial Y}+\frac{1}{\upvarepsilon }\frac{{\nu }_{nf}}{{\nu }_{f}}\frac{1}{Re}{\nabla }^{2}V-\frac{{\nu }_{nf}}{{\nu }_{f}}\frac{1}{Re}\frac{V}{Da}\frac{Fc}{\sqrt{Da}}V\sqrt{{U}^{2}+{V}^{2}}+\frac{{\beta }_{nf}}{{\beta }_{f}}Ri\theta$$

30 $$\frac{\partial \theta }{\partial \tau }+U\frac{\partial \theta }{\partial X}+V\frac{\partial \theta }{\partial Y}=\frac{{\alpha }_{nf}}{{\alpha }_{f}}\frac{1}{Re Pr}{\nabla }^{2}\theta$$

The following equation is used to measure the entropy generation^11^:

31 $${s}_{gen}= \frac{{k}_{nf}}{{T}_{0}^{2}}\left[{\left(\frac{\partial T}{\partial x}\right)}^{2}+{\left(\frac{\partial T}{\partial y}\right)}^{2}\right]+\frac{{\mu }_{nf}}{{T}_{0}^{2}}\left[2{\left(\frac{\partial u}{\partial x}\right)}^{2}+2{\left(\frac{\partial v}{\partial x}\right)}^{2}+{\left(\frac{\partial u}{\partial x}+\frac{\partial v}{\partial x}\right)}^{2}\right]+\frac{{\mu }_{nf}}{K{T}_{0}}\left({u}^{2}+{v}^{2}\right)$$

The dimensionless form of Eq. (31) is as follows:

32 $${S}_{gen}= \frac{{k}_{nf}}{{k}_{f}}\left[{\left(\frac{\partial \theta }{\partial X}\right)}^{2}+{\left(\frac{\partial \theta }{\partial Y}\right)}^{2}\right]+\chi \left\{Da\left[2{\left(\frac{\partial U}{\partial X}\right)}^{2}+2{\left(\frac{\partial V}{\partial X}\right)}^{2}+{\left(\frac{\partial U}{\partial X}+\frac{\partial V}{\partial X}\right)}^{2}\right]+\left({U}^{2}+{V}^{2}\right)\right\}$$

where $${T}_{0}=\left({T}_{h}+{T}_{c}\right)/2$$ and $$\chi ={\mu }_{nf}{T}_{0}/{k}_{nf}\left({V}_{0}/{T}_{h}-{T}_{c}\right)$$.

The t are determined from the mentioned equations in Ref.^50^. Moreover, it is worth mentioning here all governing equations for the nanofluid zone are calculated by related equations in Ref.^50^.

33 $${\rho }_{nf}=\left(1-\upphi \right){\rho }_{bf}+\upphi {\rho }_{sp}$$

34 $${({C}_{p})}_{nf}=\left(1-\upphi \right){({C}_{p})}_{bf}+\upphi {({C}_{p})}_{sp}$$

35 $${\beta }_{nf}=\left(1-\upphi \right){\rho }_{bf}+\upphi {\rho }_{sp}$$

36 $$\frac{{\mu }_{nf}}{{\mu }_{bf}}=123{\upphi }^{2}+7.3\upphi +1$$

37 $$\frac{{k}_{nf}}{{k}_{bf}}= \frac{{k}_{sp}+2{k}_{bf}-2\upphi ({k}_{bf}-{k}_{sp})}{{k}_{sp}+2{k}_{bf}+\upphi ({k}_{bf}-{k}_{sp})}$$

To calculate the angular velocity and its magnitude, a simple harmonic motion equation is used, which is defined as follows:

38 $$x\left(t\right)=A\mathrm{cos}\left(\frac{2\pi }{T}t\right)$$

The local and average Nusselt numbers are defined as follows:

39 $$Nu= -\left(\frac{{k}_{nf}}{{k}_{f}}\right)\frac{\partial \theta }{\partial X}$$

40 $$Nu=\underset{0}{\overset{1}{\int }}{\left.Nudy\right|}_{x=0}$$

The dimensionless boundary conditions are as follows,

**Table:** 

Coordinates			
Y = 1	u = V_0_, v = 0, θ = 0	Top wall	(41)
Y = 0	u = v = 0, θ = 1	Bottom wall	(42)
X = 0, X = 1	u = v = 0, $\frac{\partial \theta }{\partial X}=0$	Vertical walls	(43)
X = Y = 0.35	u = Ω(Y), v = Ω(X), $\frac{\partial \theta }{\partial n}=0$	Rotating cylinder wall	(44)

The interface between fluid and the permeable zone is considered as follows,

45 $$\frac{{\partial \theta }_{nf}}{\partial n}=\frac{{\partial \theta }_{porous}}{\partial n}, \frac{{\partial {\omega }^{*}}_{nf}}{\partial n}=\frac{{\partial {\omega }^{*}}_{porous}}{\partial n}, \frac{{\partial {\uppsi }^{*}}_{nf}}{\partial n}=\frac{{\partial {\uppsi }^{*}}_{porous}}{\partial n}$$

## Numerical method

The finite volume method (FVM), which maintains fluxes balance between adjacent control volumes, was implemented to solve the conservation equations of mass, momentum, and energy. A pressure-based solver was applied to solve the flow fields. The second-order upwind scheme method was used for interpolation in the transport equations. With this method, a higher order of accuracy in each aspect of the cell was achieved through a Taylor series expansion for solving the cell center information. The second-order upwind method was been used to interpolating the pressure. The SIMPLE algorithm, which is a semi-implicit method, was used for pressure-related equations. The convergence criterion of 10^–7^ was considered for the above-mentioned equations^53^.

## Grid study and validation

**Figure 2:**
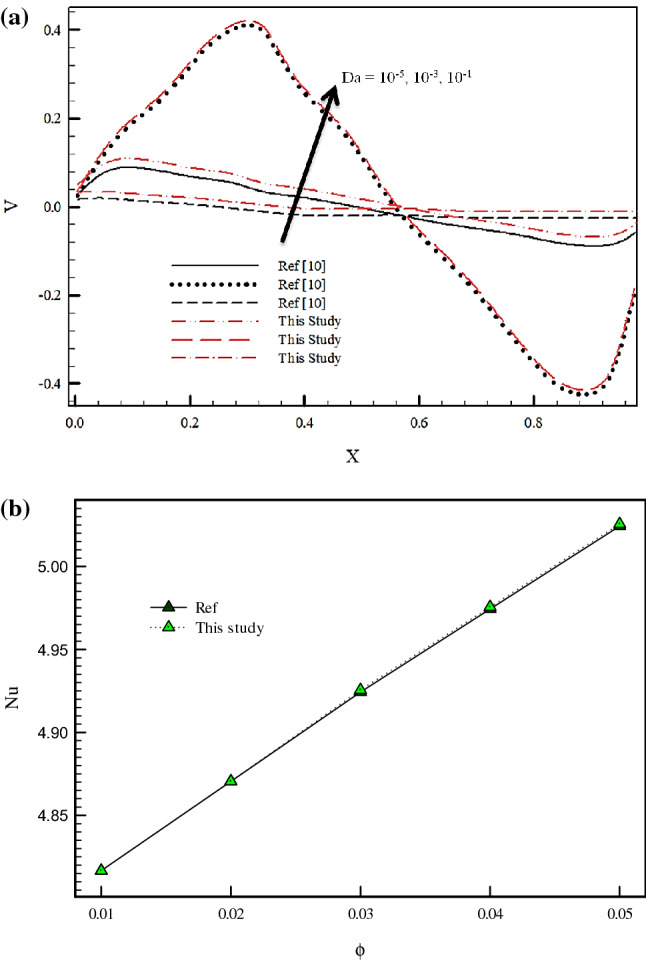
(**a**) Validation of present numerical code with Ref.^10^. (**b**) Validation of present numerical code with Ref.^20^.

**Figure 3:**
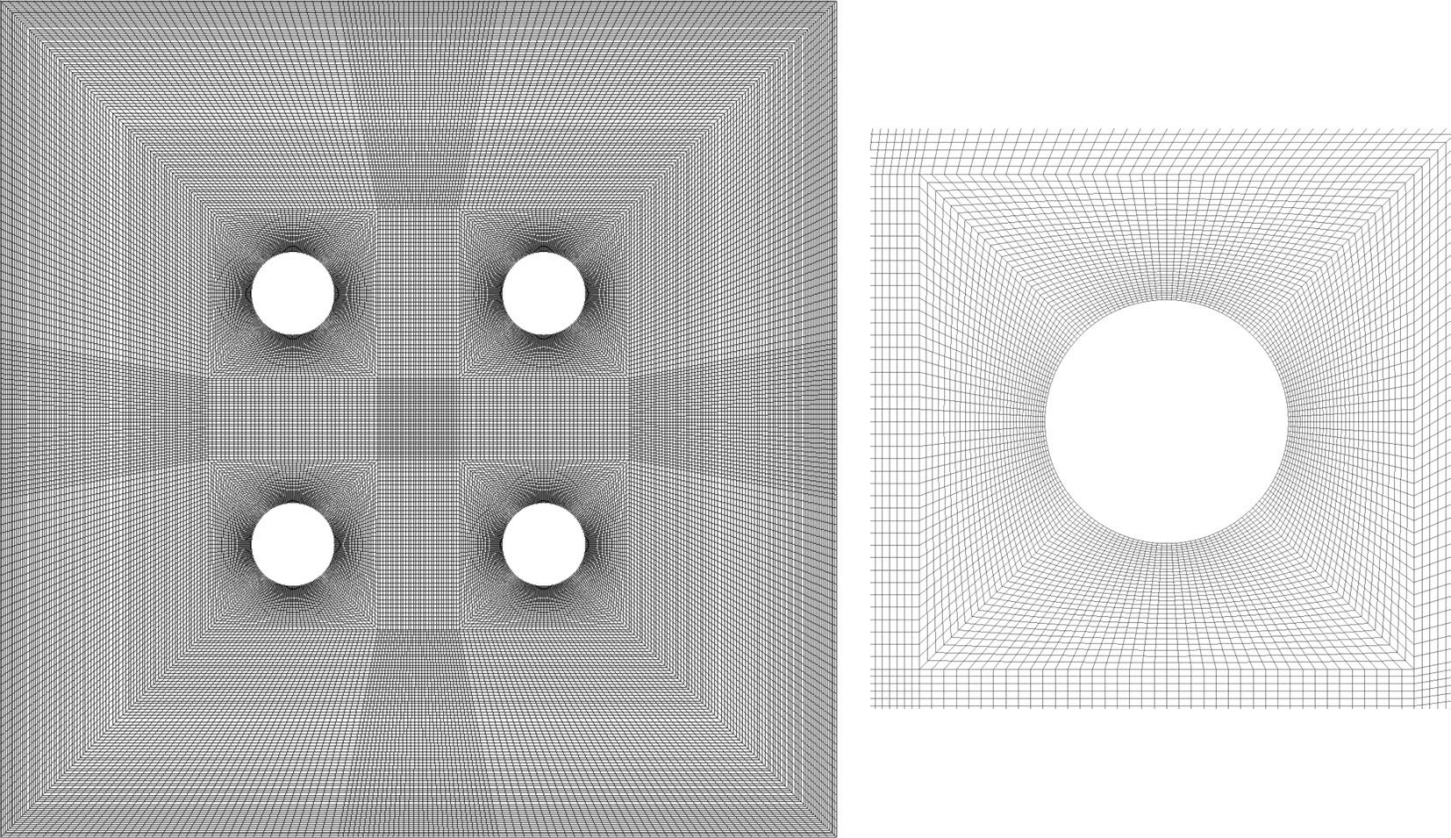
Mesh structure.

In numerical studies, validation was used to ensure the selected parameters and solution method in the computer code. To validate the accuracy of our in-house numerical code, a test case was performed. The validation of the present study was verified against the reported results by Rajarathinam et al.^10^. The results of this comparison were shown in Fig. 2. As can be seen, there was an acceptable agreement between this study and Ref.^10^. Moreover, to further ensure the setups for the rotating cylinder, a comparison was made with Ref.^20^ in Fig. 2b. Figure 3 shows the used grid in this study. The grid study test (Table 2) was performed by comparing the Nusselt number for Ri = 1 and ϕ = 0.01. The grid structure with 60,850 elements and 30,882 nods was selected to solve this problem owing to its minor variance of Nusselt number.

**Table 2:** Grid independency test.

Cell number	Nu_ave_
30,956	10.4524
44,998	10.4882
60,850	10.4901
80,568	10.4996

## Results and discussion

**Figure 4:**
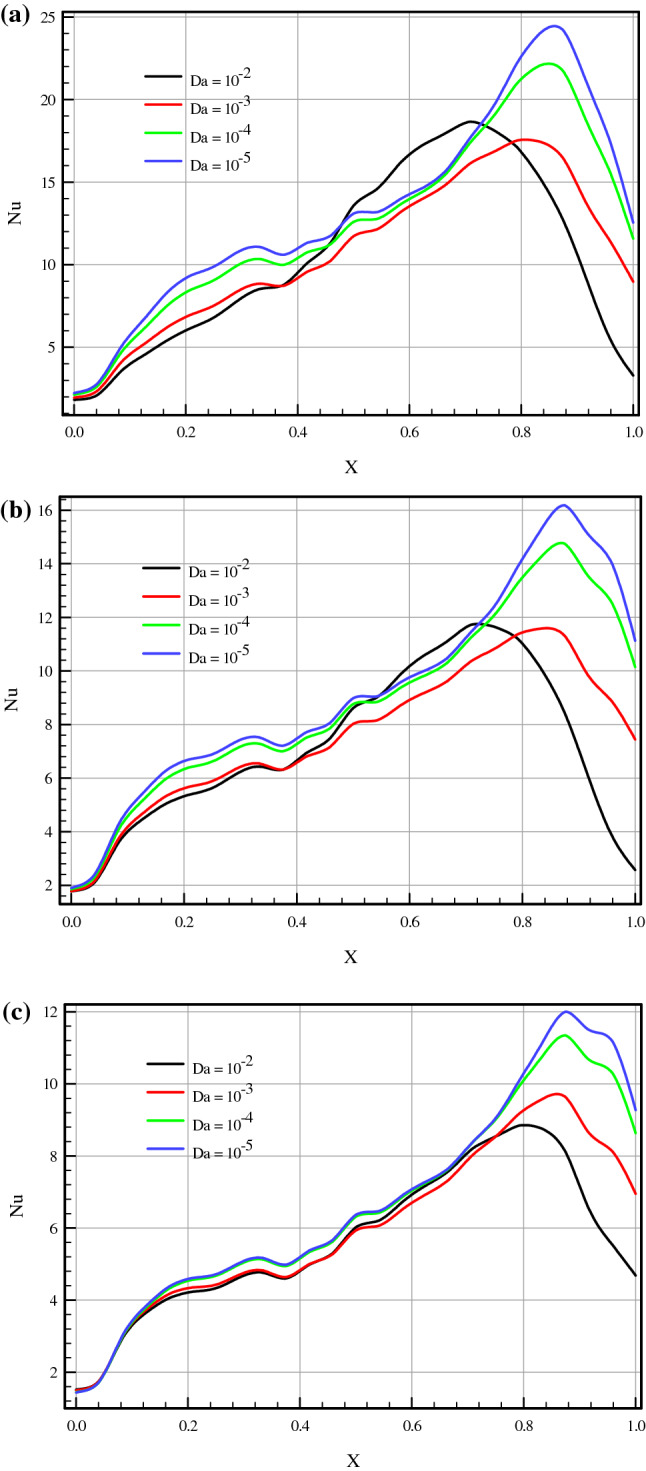
(**a**) Nusselt number along lower wall for Ri = 0.1, ɛ = 0.5, τ = 0.1. (**b**) Nusselt number along lower wall for Ri = 1, ɛ = 0.5, τ = 0.1. (**c**) Nusselt number along lower wall for Ri = 10, ɛ = 0.5, τ = 0.1.

**Figure 5:**
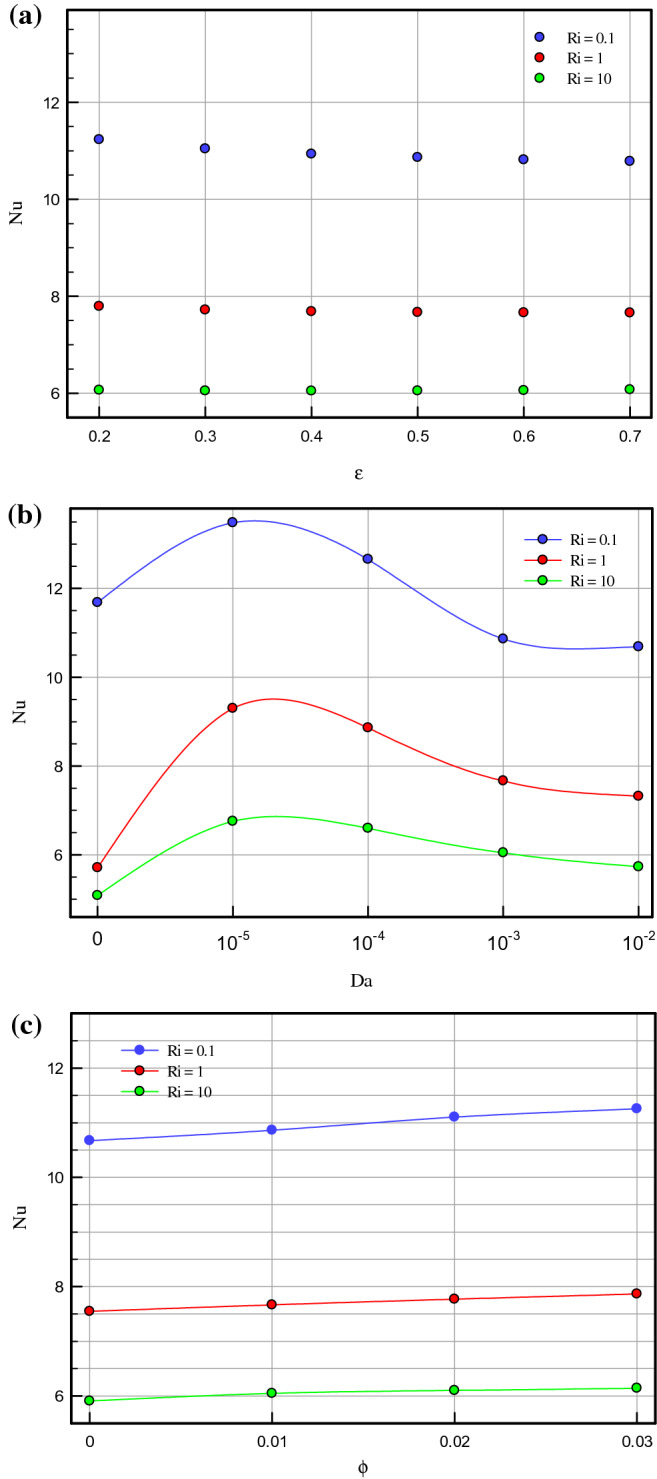
(**a**) Average Nusselt number for Da = 10^–3^, ϕ = 0.01, τ = 0.1. (**b**) Average Nusselt number for ɛ = 0.7, ϕ = 0.01, τ = 0.1. (**c**) Average Nusselt number for Da = 10^–3^, ɛ = 0.5, τ = 0.1.

**Figure 6:**
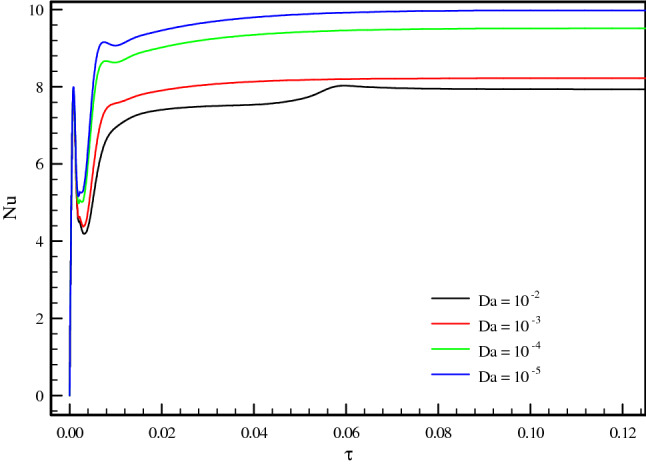
Nusselt number variation over the time for ϕ = 0.01, Ri = 1, and ɛ = 0.7.

**Figure 7:**
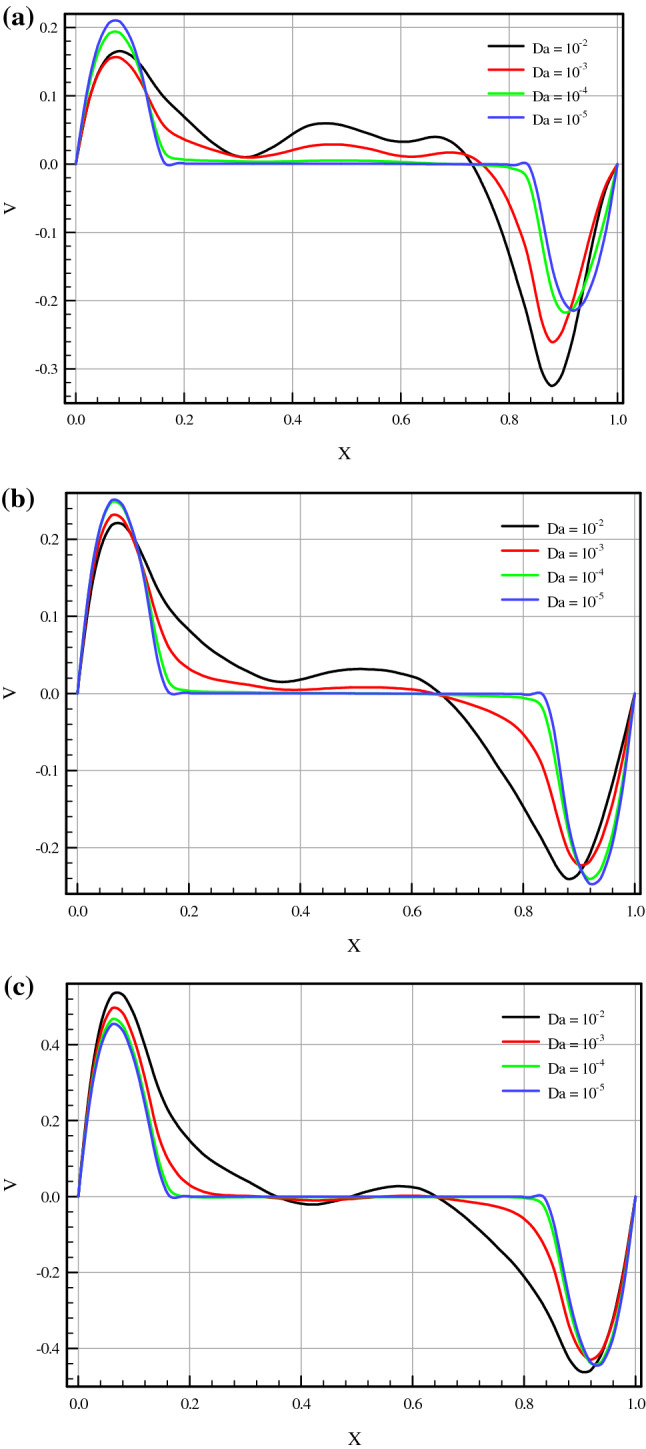
(**a**) Effect of different Darcy number on vertical velocity for τ = 0.1, ϕ = 0.03, Ri = 0.1, and ɛ = 0.5. (**b**) Effect of different Darcy number on vertical velocity for τ = 0.1, ϕ = 0.03, Ri = 1, and ɛ = 0.5. (**c**) Effect of different Darcy number on vertical velocity for τ = 0.1, ϕ = 0.03, Ri = 10, and ɛ = 0.5.

**Figure 8:**
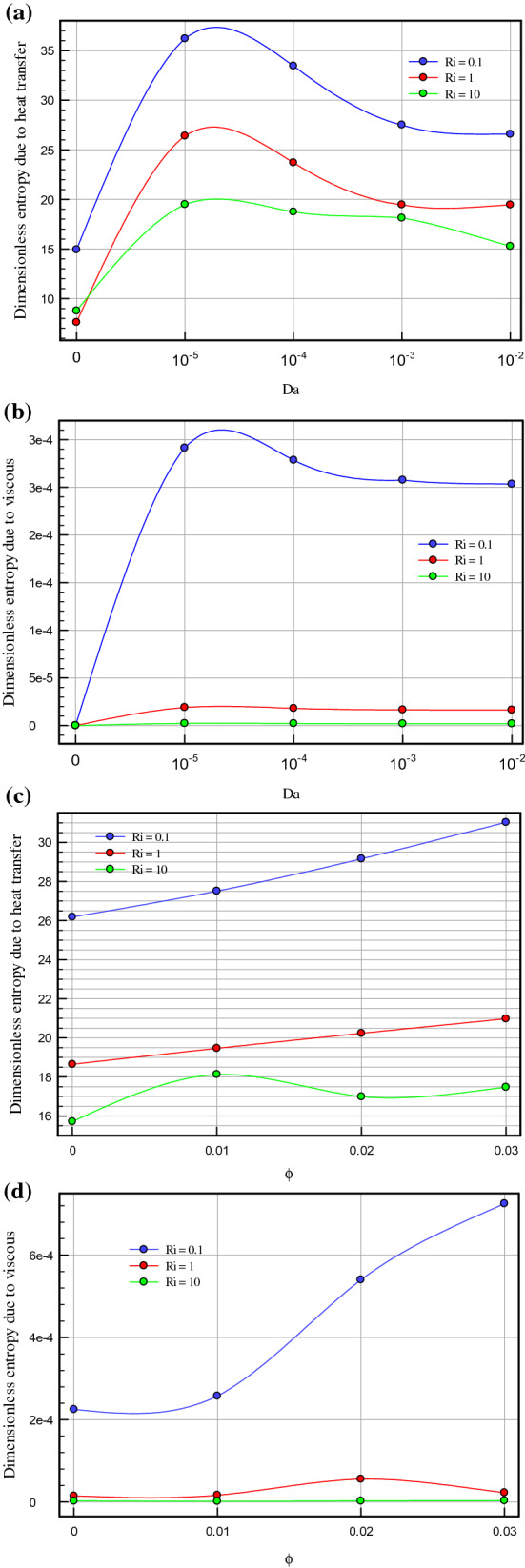
(**a**) Effect of different Darcy number on entropy generation for τ = 0.1, ϕ = 0.03, ɛ = 0.7. (**b**) Effect of different Darcy number on entropy generation for τ = 0.1, ϕ = 0.03, ɛ = 0.7. (**c**) Effect of different Darcy number on entropy generation for τ = 0.1, ɛ = 0.7, Da = 10^–3^. (**d**) Effect of different Darcy number on entropy generation for τ = 0.1, ɛ = 0.7, Da = 10^–3^.

**Figure 9:**
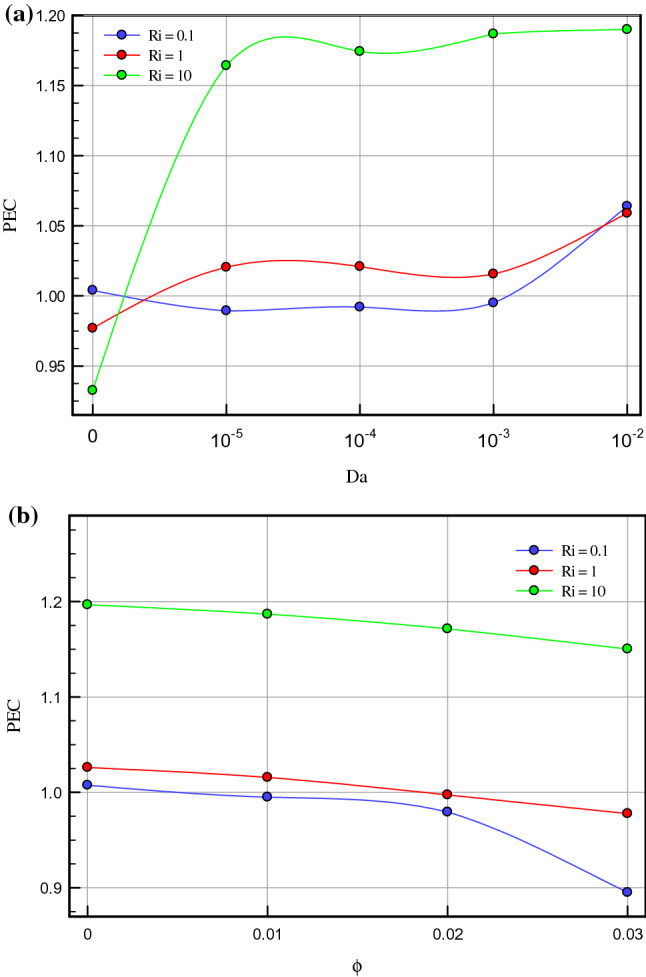
(**a**) Effect of different Darcy number on PEC for τ = 0.1, ϕ = 0.03, ɛ = 0.7. (**b**) Effect of different volume fraction of nanoparticles on PEC for τ = 0.1, Da = 10^–3^, ɛ = 0.5.

**Figure 10:**
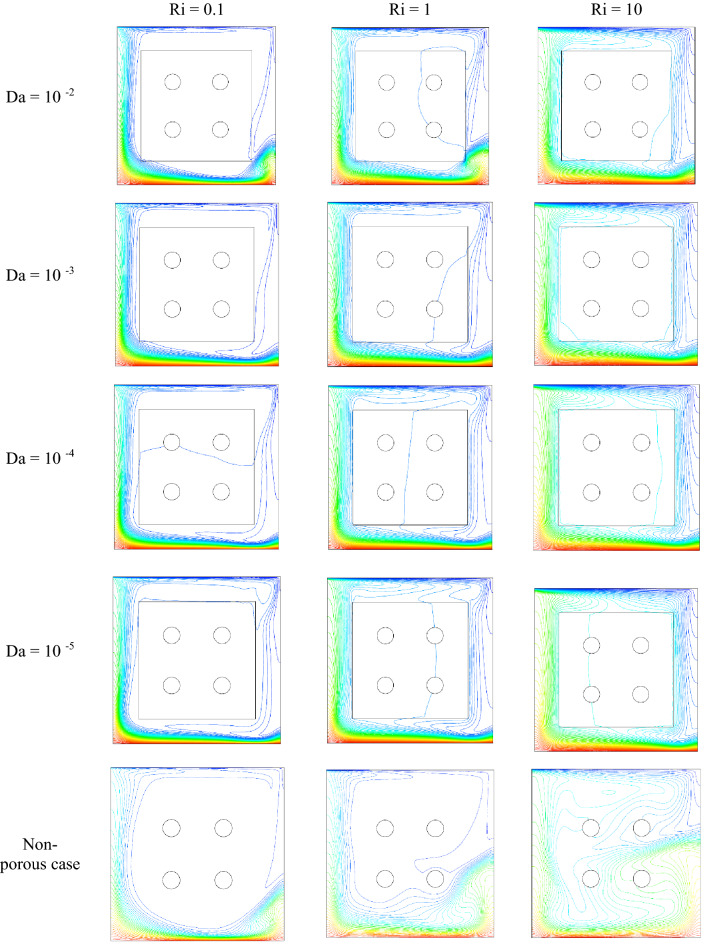
Dimensionless Isotherm contours for φ = 0.01, τ = 0.1, ɛ = 0.5.

**Figure 11:**
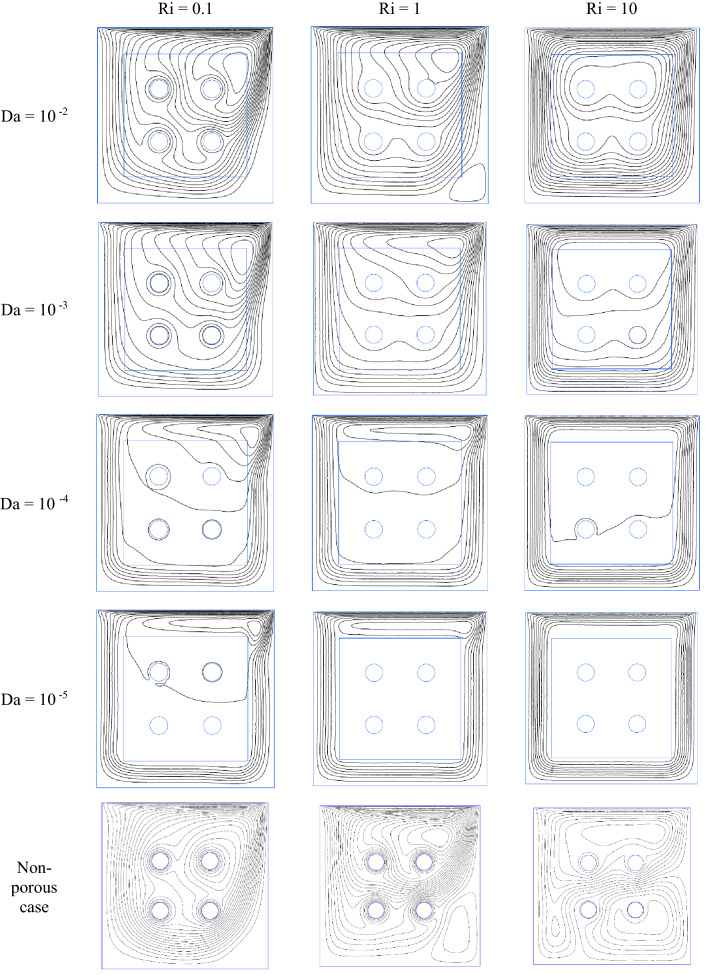
Dimensionless Streamline contours for φ = 0.01, τ = 0.1, ɛ = 0.5.

Numerical investigation was conducted for Darcy number (10^–5^ ≤ Da ≤ 10^–2^), porosity (0.2 ≤ ɛ ≤ 0.7), Richardson number (0.1 ≤ Ri ≤ 10), Prandtl number (Pr = 6.196), Grashof number (Gr = 74,363.3974), and dimensionless angular velocity (Ω = 5). Streamline contours, isotherm contours, local and average Nusselt numbers, dimensionless velocity, entropy generation, and PEC for different control characteristics mentioned above were presented in Figs. 4, 5, 6, 7, 8, 9, 10, and 11.

Figure 4a–c demonstrate the local Nusselt number on hot wall for various values of Darcy number (Da = 10^–2^, 10^–3^, 10^–4^, and 10^–5^) and Richardson number (Ri = 0.1, 1, and 10). As can be seen in the line graphs, for all Richardson numbers, the highest point of the Nusselt number graphs displaces in the vicinity of the right wall of the cavity at dimensionless length between X = 0.8 and X = 1.0. This intensification in the Nusselt number on the right side of the cavity can be attributed to the driven flow from the lid. However, the values of the Darcy number have a positive contribution towards the Nusselt number. By declining the Darcy number, the flow makes a shift to a non-Darcy flow regime. If one considers the difference in the behavior of local Nusselt number diagrams related to higher and lower values of the Darcy numbers, one can perceive the dominance of non-Darcy flow in lower values of Darcy numbers (Da = 10^–4^ and 10^–5^). By decreasing Darcy number from Da = 10^–2^ to Da = 10^–5^, the peak value of Nusselt number for Ri = 0.1, 1 and 10 increase by 40.651%, 43.32% and 49.95%, respectively. Figure 5a–c are illustrate the effect of various porosity (ɛ = 0.2, 0.3, 0.4, 0.5, 0.6, and 0.7), Darcy number (Da = 0, 10^–5^, 10^–4^, 10^–3^, 10^–2^), and volume fraction of nanoparticle (ϕ = 0, 0.01, 0.02, and 0.03) on local Nusselt number at hot wall of square cavity, in turn. As illustrated in Fig. 5a, changes in the average Nusselt number on the hot wall for porosity changes are negligible. However, when porosity rises from 0.2 to 0.4, the value of the Nusselt number sees a marginal drop which is more recognizable for mixed and forced convection. A decrease in Richardson number leads to decreasing buoyancy forces and consequently enhancing heat transfer rate. In this case, a decrease in Richardson number from 10 to 1 and 1 to 0.1 increases the average Nusselt number by about 30% and 44.87%, respectively. To study the impact of variegated Darcy numbers on the average Nusselt number on the hot wall Fig. 5b is presented. It can be seen from the graph that the existence of a centered porous block in the square cavity has a positive influence on the Nusselt number. The maximum number of the Nusselt number is for Da = 10^–5^ where momentum diffusion governs in the porous zone. While the Darcy number is lower, permeability is also low; ergo, the velocity and temperature gradients in the middle of the chamber are minor. Hence, the driven flow circulates faster along the boundaries which resulting in better enclosure cooling. As Richardson's number decreases, the flow regime is overwhelmed by shear forces which cause a high flow rate in the cavity, and subsequently, the heat transfer rate enhances. Decreasing the Richardson number from 10 to 0.1 with a fixed Darcy number escalations the average Nusselt number by about 91.6% due to the dominance of mechanical effects from lid-driven and cylinders with harmonic motion. Figure 5c compares the effect of accumulation of volume fractions of nanoparticles upon average Nusselt number on the hot wall. The most marked feature of the chart is the growth of the average Nusselt number with adding solid volume fraction which is caused by the enhanced thermal conductivity of nanofluid. However, with the dominance of natural convection, the influence of increasing the volume fraction of nanoparticles decreases. An augmentation in nanoparticle volume fraction leads to enhancing Nusselt number by 7.351%, 7.965%, and 9.32% for Ri = 10, 1, and 10, respectively. By comparing the diagrams, it can be seen that at high Richardson numbers, where the fluid momentum is low and the flow layers adjacent to the walls are not less concentrated than at lower Richardson numbers, the Nusselt number is smaller. Moreover, due to significant velocity gradient changes near the cylinders, the impact of rotational cylinders having harmonic motion is more evident on heat transfer rate at Ri = 0.1. To illustrate the point, for the same geometry, if the cylinders remain motionless, the heat transfer rate drops by 16.41%. Figure 6 compares the average Nusselt number for different Darcy numbers versus dimensionless time. Although the figure shows the dimensionless time from 0 to 0.12, most cases are calculated up to τ = 0.35 to be sure of changes over time. Nevertheless, we only considered the results at τ = 0.1, because in all cases, the solution reaches a steady-state after this time. As can be seen from this figure, in the early stages of solving, the Nusselt number diagrams experience oscillations that could be due to the effect of changes in the mainstream, which is driven by the moving wall and vortices on the thermal boundary layers. From τ = 0.07 onwards, all fluctuation are damped and figures remain independent of time changes and depend on the Darcy number. Among the compared Darcy numbers, the Da = 10^–2^, which is less permeable and the boundary layer is more affected by the rotational flow around the porous block, takes more time to stabilize the Nusselt number. Figure 7a–c are illustrate the impact of various Darcy numbers on vertical velocity profiles on the midline of the cavity for τ = 01, ϕ = 0.03, ɛ = 0.5, and Ri = 0.1, 1, and 10. As shown in the graphs, the greatest magnitudes of vertical velocity for all tested cases are observed at the non-porous zone. The magnitude of velocity in this region is mostly related to driven flow from the upper moving walls and emerged vortex in different parts of the cavity; nonetheless, higher values of permeability velocity profiles are also partially affected by the porous region. In the porous zone velocity almost have an equal magnitude in particular for Da = 10^–4^ and 10^–5^. As Darcy number decreases, velocity profiles in the porous zone are affected by driven flow from cylinders with harmonic motion and it is more distinguishable for mixed and forced convection where the flow is overwhelmed by shear forces. Due to the extreme fluctuations of the fluid flow, which are caused by momentary stop and change the direction of rotation of the cylinders, changes in the velocity profile in the porous region are unpredictable. Due to the lower resistance in the region with a higher Darcy number, the velocity profile has the highest value for this range of Darcy numbers (Da = 10^–2^ and 10^–3^). Figure 8a–d show the effect of various Darcy numbers and volume fraction of nanoparticles on entropy generation. It is worth mentioning here that entropy generation due to viscosity and heat transfer is dispensed in dimensionless form. According to the second law of thermodynamics, all or part of the exergy that is proportional to the production of entropy disappears. Entropy is studied to determine the reversibility or irreversibility of a process in a system. For all figures, the increase of the Richardson number reduces entropy generation owing to the reduction in shear rate and the predominance of free convection. Moreover, increasing the nanoparticles improves the viscosity of the fluid and as a result, increases the entropy generation; however, as the Richardson number magnifies and the resulting inertial forces decrease, the effects of increasing the nanoparticle volume fraction in this comparison become negligible. One can notice from the figure that entropy generation number due to viscosity effects are much less than entropy generation caused by heat transfer; therefore, its effect is negligible due to small velocity gradients in presence of porous zone. By comparing the figures, it can be seen that with decreasing pore diameter, which leads to decreasing permeability, the number of entropy generation due to both heat transfer and viscosity effects increases. The total entropy generated increases maximum by 26% and minimum 18.5% for decreasing the Darcy number and also by increasing the volume fraction of solid particles from 0.01 to 0.03, the number of total entropy generation for Ri = 0.1 increases by about 19%. Figure 9a and b illustrate changes in performance evaluation criteria (PEC) for different volume fractions of nanoparticles and Darcy numbers. The PEC parameter compares the Nusselt number and the coefficient of friction at the heated wall. This parameter is a measure of the heat transfer enhancement to pressure drop increment applied to the system. When the increase in heat transfer overcomes the pressure drop augmentation, this number grows to more than one, which indicates the system is cost-effective. As can be seen in the graphs, bolstering buoyancy forces leads to escalating the PEC parameter. As Darcy number increases from 10^–5^ to 10^–2^, the PEC parameter for Ri = 0.1, 1, and 10 increase 12.43%, 18.95%, and 23.43%, respectively. Moreover, adding nanoparticle to base fluid from 0.01 to 0.03 leads to dropping PEC value of 17.43%, 12.01%, and 10.63% for Ri = 0.1, 1, and 10, in turn. Figures 10 and 11 present a detailed comparison of isotherms contours and streamlines contours for variegated Darcy number, φ = 0.01, τ = 0.1, ɛ = 0.5. It can be seen from the isotherms contours that for a fixed value of Darcy number by increasing Richardson number the temperature gradients spread around the porous zone. On the contrary, by reducing the effect of buoyancy forces, the thermal layers are concentrated at the bottom of the enclosure. Moreover, for higher Darcy number and lower Richardson number minor temperature gradients occurs in the porous zone. However, this is verifiable for Da = 10^–4^ and Ri = 0.1. The presence of a high-temperature thermal layer as well as a denser thermal layer formed near the wall for Ri = 0.1 is the reason for the improvement of heat transfer in forced convection. As the Darcy number decreases, the thermal boundary layers become thinner. Moreover, as the permeability decreases (ergo less fluid penetration in the porous zone) more cold thermal plumes sway to the right of the chamber which helps to enhance the cooling process. As can be seen from streamline contours, the movement of the lid generates a clockwise rotational flow which is also affected by the harmonic movement of the cylinders and the permeability of the porous medium. Owing to the dominance of mechanical effects, a vortex, which skewed to the left half of the cavity with decreasing Darcy number, is developed at the upper right half of the cavity for Ri = 0.1 and 1. Decreasing in Darcy number means decreasing permeability; hence, for Ri = 10, for the lower value of Darcy number, the streamlines are circulated by a single clockwise flow around the porous zone. In contrast, when the Darcy number increases the flow rate permeates in the porous zone where is affected by the harmonic motion of cylinders. For these cases, one can notice the uneven streamlines in the porous zone owing to cylinders effects. Nonetheless, for Ri = 10, which shear forces suppressed by buoyancy force and conduction-dominated regime, streamlines in the porous block become more even.

## Conclusion

In conclusion, mixed convection of Cu-water nanofluid flow in a cavity with porous block and rotational cylinders was numerically investigated in the Finite Volume Method. The porous zone was studied considering the Forchheimer–Brinkman-extended Darcy model. A simple harmonic function has been considered for the direction of rotation of the cylinders. The results demonstrate the presence of porous blocks in this geometry produces a positive effect on the heat transfer rate. However, this desired effect is more discernible for lower Darcy numbers. That is, by decreasing Darcy number from 10^–2^ to 10^–5^ the Nusselt number increases by 43.32% for Ri = 1. Moreover, the highest value of dimensionless entropy generation and PEC number, as well as Nusselt number, is for Da = 10^–5^. The effect of the harmonic rotation of the cylinders on flow patterns becomes more pronounced when the permeability of the porous medium is higher. In this study, the decrease in Richardson numbers from 10 to 0.1 resulted in a 76.92% increase in heat transfer rate. The presence of nanoparticles also increased the average Nusselt number by up to 30%, which the percentage of change decreases with increasing Richardson number.

## Greek signs

β: Thermal expansion coefficient (1/K)
θ: Dimensionless temperature
μ: Viscosity (kg/ms)
ρ: Density (kg/m^3^)
τ: Dimensionless time
ϕ: The volume fraction of solid particle
χ: Reversibility function
ψ: Stream function (m^2^/s)
Ω: Dimensionless angular velocity
${\omega }_{0}$: Angular velocity (rad/s)
$$\varepsilon$$: Porosity

## Subscript

c: Cold
h: Hot
bf: Base fluid
nf: Nanofluids
sp: Solid particles
p: Particle

## Latin symbols

C_p_: Specific heat capacity(J/kg K)
g: Gravity (m/s^2^)
h: Heat transfer coefficient (W/m^2^ K)
L: Cavity length (m)
k: Thermal conductivity (W/m K)
Nu: Nusselt number
p: Pressure (Pa)
Ri: Richardson number
Re: Reynolds number
s_gen_: Entropy generation (W/m^3^K)
S_gen_: Dimensionless entropy generation
T: Temperature (K)
u, v: Velocity components (m/s)
U_0_: Dimensionless velocity of moving wall
U, V: Dimensionless velocity components u/U_0_, v/U_0_
x, y: Cartesian coordinates (m)
X, Y: Dimensionless Cartesian coordinates x/H, y/H
Da: Darcy number
PEC: Performance Evaluation Criterion
f: Friction factor
Gr: Grashof number
Pr: Prandtl number
$Cf$: Surface friction factor
